# Niagara University

**Published:** 1884-07

**Authors:** 


### Niagara University.

We learn that the medical department of this University has acquired possession of the property on Ellicott street near the corner of Broadway, and immediately opposite to the site upon which will be erected the magnificent buildings for the Young Men's Association and Grosvenor libraries. A more eligible site for the new college could not have been found in the city; not only is it convenient to the hospitals and dispensaries, but also, all of the scientific societies will have their home in the new library buildings, and the proximity of their museum and collection will be of advantage to the students of the Niagara College. A greater advantage, however, will be the ability to make free use of the Grosvenor library and its splendid collection of books of reference. The present location of this library is but half a block away, so that its advantages will be available the coming winter.

The new college building, which it is proposed to immediately erect upon this property, will be ready for occupancy in October. Plans of the building have been submitted by several noted architects, and we are informed that in the one adopted the comfort and convenience of the students will be constantly borne in mind in the arrangement of its rooms, lecture halls and laboratories.

